# Preventing bacterial disease in poultry in the post-antibiotic era: a case for innate immunity modulation as an alternative to antibiotic use

**DOI:** 10.3389/fimmu.2023.1205869

**Published:** 2023-07-03

**Authors:** James R. G. Adams, Jai Mehat, Roberto La Ragione, Shahriar Behboudi

**Affiliations:** ^1^ School of Veterinary Medicine, Faculty of Health and Medical Sciences, University of Surrey, Guildford, United Kingdom; ^2^ Avian Immunology, The Pirbright Institute, Woking, United Kingdom; ^3^ School of Biosciences, Faculty of Health and Medical Sciences, University of Surrey, Guildford, United Kingdom

**Keywords:** immune modulators, chickens, avian bacterial pathogens, prebiotics and probiotics, pathogen associated molecular patterns (PAMPs), microbial metabolites and derivatives, vitamins

## Abstract

The widespread use of antibiotics in the poultry industry has led to the emergence of antibiotic-resistant bacteria, which pose a significant health risk to humans and animals. These public health concerns, which have led to legislation limiting antibiotic use in animals, drive the need to find alternative strategies for controlling and treating bacterial infections. Modulation of the avian innate immune system using immunostimulatory compounds provides a promising solution to enhance poultry immune responses to a broad range of bacterial infections without the risk of generating antibiotic resistance. An array of immunomodulatory compounds have been investigated for their impact on poultry performance and immune responses. However, further research is required to identify compounds capable of controlling bacterial infections without detrimentally affecting bird performance. It is also crucial to determine the safety and effectiveness of these compounds in conjunction with poultry vaccines. This review provides an overview of the various immune modulators known to enhance innate immunity against avian bacterial pathogens in chickens, and describes the mechanisms involved.

## 1 Introduction

By 2050, the world’s population is predicted to increase to 9.8 billion people, threatening food security and causing shifts in consumer purchasing patterns (1, 2). Animal-derived meat products are central to the current food supply, accounting for 40% of the agricultural production value and 13% of the calories consumed globally. Furthermore, based on consumer patterns meat consumption is predicted to increase by between 62% to 144% by 2050, with consumption of poultry alone predicted to double (2, 3). To accommodate this increased demand, the poultry industry must expand beyond its current size or innovate its current production methods. However, accommodating this increased demand incurs greater costs, difficulty in maintaining production yields, meat quality issues, and animal welfare concerns (4, 5). Infectious diseases such as mycoplasmosis and laryngotracheitis are significant contributors to these factors, with increased production intensity resulting in the emergence of diseases and antimicrobial resistance (AMR). Jones et al. (2019) observed that diseases associated with production, namely respiratory diseases/conditions (ascites and infectious bronchitis), enteric diseases (coccidiosis and clostridiosis), locomotory diseases (tibial dyschondroplasia, foot pad dermatitis, and keel bone damage), colibacillosis affecting reproduction, and injurious feather pecking, can increase bird mortality by up to 336% in broiler flocks and 71.5% in layer flocks (6). These conditions were also observed to reduce productivity, with broiler body weight reduced by as much as 17.7%, and egg numbers and egg weight reduced by 32.9% and 8.7%, respectively (6). The incidence of diseases within poultry flocks can stem from a range of sources, including viruses, parasites, and bacteria, with the latter being the focus of this review.

To ensure productivity and maintain animal welfare, mitigation of the emergence and spread of bacterial diseases is essential. Current management strategies are focused on biosecurity, antibiotics, and vaccines. Biosecurity broadly refers to the measures taken to prevent the introduction or minimize the spread of infectious diseases (7). The elimination of pathogenic bacteria such as *Salmonella* within flocks can pose a significant challenge because of their ubiquitous nature. However, the application of sanitation technologies, combined with effective surveillance, prevention, and management strategies can result in the reduction or eradication of epidemiologically important human pathogens, such as *Salmonella* Typhi, in higher income countries (8). In poultry, sanitation strategies typically aim to reduce contamination of feed, water, and hatching eggs, as these are frequently seen as common points of disease transmission (9). These strategies include improving the cleanliness of the hatching area and sanitization of eggs using disinfectants or UV radiation (9, 10). Improvements in feed and water supply by pasteurization and chlorination allow the number of pathogenic strains within the gastrointestinal tract and feces to be reduced in a non-invasive manner, limiting the spread of bacterial diseases such as colibacillosis (11, 12). Improvement in air and litter quality have also been demonstrated to reduce the risk of outbreaks, highlighting the importance of environmental management (13).

Antibiotics have historically been applied on a flock-wide scale to prevent and treat bacterial diseases, as well as to promote bird growth and egg production (14). Tetracyclines, aminoglycosides, and fluoroquinolones are among the antibiotic classes approved for use by regulatory authorities in the UK, Brazil, China, and Europe, and where permitted can be employed prophylactically (14, 15). Glycolipids, macrolides, and glycopeptides are used as growth-promoting antibiotics (16) but have been prohibited for use in agriculture in the EU since 2006, with recent legislation further restricting their use (17, 18). Similar legislative efforts and antibiotic stewardship campaigns have been seen globally, including in the US, Japan, Denmark, China, and India (19, 20). However, the use of agricultural antibiotics remains prevalent in low- and middle-income countries because of their reliance on food and animals as exports, as well as food insecurity (20). The historic, current, and misuse of antibiotics have led to the selection and promotion of antimicrobial resistance (AMR) within bacterial populations. The presence of antimicrobials within an environment following misuse results in selection pressure, eliminating susceptible organisms and allowing those with acquired or intrinsic resistance to survival and multiplication (21). The selection of AMR, particularly within populations associated with infectious diseases, is regarded as one of the most pressing issues facing global public and animal health (14, 22, 23).

In addition to antibiotics and improved sanitation, several types of vaccines are employed to protect against viral, parasitic, and bacterial pathogens, including live attenuated, inactivated/killed, nucleic acid-based, and subunit vaccines (24, 25). Autogenous vaccination, generated by the isolation and inactivation of bacteria from diseased animals for immunization of flocks, has also been frequent use, but its use is now restricted within the EU to emergency cases when licensed vaccines are available, thus their usage may change in the future (26). Vaccination also suffers from a lack of long-term efficiency due to genomic diversity and drift within pathogens, leading to reduced protection (25, 27). Furthermore, issues have also been identified with the administration of vaccines, with some requiring multiple doses and uneven application resulting in reduced efficacy (28). Administration-associated issues can be mitigated through the application of vaccines through spray, orally, or *in ovo* (29), but the specificity of vaccination still demands the application of multiple vaccines, each targeting an individual pathogen or pathogen group, driving up costs, and increasing the labor required for production (24, 27).

This growing desire for non-antibiotic treatments has led to an increased interest in alternative control strategies. Bacteriophage therapy, the use of viruses to control and treat bacterial pathogens, has seen greater focus, with over a dozen bacteriophage products for use in poultry coming to market, with many receiving “generally recognized as safe” certification by the US Food and Drug Administration (30). Furthermore, a range of non-antibiotic methods have also been employed to manage infectious diseases, such as pre- and probiotics (31), plant-derived phytochemicals (32), and organic or short-chain fatty acids (33), many of which have been observed to directly inhibit the growth of pathogenic bacteria. However, bacterial diseases remain a key issue in poultry production and may cripple poultry production in the future. Therefore, novel management strategies to supplement or replace existing methods are required. Notably, the ability to generate sustained heterologous protection through minimal application, while avoiding AMR selection is strongly desired. A proposed alternative to the use of antibiotics and traditional vaccination is to target the innate immune system using immunostimulatory compounds.

## 2 Overview of the avian immune system

### 2.1 Innate immune system

Like most vertebrates, the avian immune system consists of a rapid non-specific innate immune system and a highly specialized adaptive immune system (34). The avian innate immune system consists of a broad range of physiological barriers as well as effector cells that generate rapid broad-spectrum responses encoded by germline genes. Several functionally distinct effector cells are present within the avian innate immune system, including natural killer (NK)-cells, heterophils (the avian homologue of neutrophils), macrophages, and innate-like T cells (e.g., γ-δ T cells), which were explored in detail here (35).

Avian monocytes are the major phagocytic component of the blood, which originate from bone marrow stem cells (BMSCs) and can differentiate into monoblasts, pro-monocytes, and monocytes. Avian monocytes can further differentiate into macrophages which populate and reside in different tissues (36). In contrast to mammals, the avian respiratory system lacks alveoli and a resident respiratory macrophage population, with lung lavages of healthy birds rarely being able to recover immune cells (36, 37). In birds, highly phagocytic free avian respiratory macrophages (FARMs) with effective antimicrobial activity appear to be key factors in pulmonary defense (38). FARM rapidly translocates to the lung following challenge, with lung macrophage population seen to increase dramatically following avirulent *Pasteurella multocida* intratracheal immunization (39), as well as *E. coli* and *Salmonella challenge* (36, 39). Induction of macrophage recruitment has been shown to provide non-specific protection, with intratracheal dosing of chickens with *P. multocida* Chloral vaccination strain protecting against virulent *E. coli* air-sac challenge 7 h later (40). This protection is attributed to the increased recruitment of macrophages to the site of infection or induction of innate immune memory.

The phagocytic function of avian macrophages is well characterized, and internalization has been demonstrated to increase when targets have been opsonized, suggesting a key role of receptors in this process, with Fc-υ and complement receptors for IgY and C3 opsonization, respectively, suggested to be significant (36). In addition, macrophages are activated by pattern recognition receptors (PRRs) that recognize pathogen-associated molecular patterns (PAMPs), including lipopolysaccharides (LPSs), flagellin, and foreign nucleic acids, triggering a physiological or signaling response (35, 41).

Toll-like receptors (TLRs) are the most studied class of PRR and, upon stimulation, activate the NF-κB and type I interferon (IFN) pathways (42). These release signaling molecules, including inflammatory cytokines and chemokines, as well as type I IFNs, leading to the recruitment of other immune cells (35). In total, 13 TLRs have been identified in mammalian species, of which orthologues of six have been found in chickens, alongside four additional TLRs. Chicken TLRs (chTLRs) 3, 4, 5, and 7, as well as duplicated chTLRs 2A and 2B, are direct orthologs of mammalian TLRs of the same name. Furthermore, the chicken repertoire also includes chTLR 21, an ortholog of TLR 21 in fish and amphibians, and chTLR 1LA, 1LB, and 15, which have only been reported in avian species (43). Several TLRs are associated with the response to bacteria, with disruption of chTLRs 1, 2, 4, 5, 7, 15, and 21 each of which associated with susceptibility to *E. coli* and *Salmonella* infection (43, 44).

### 2.2 Adaptive immune system

In contrast to the low specificity of the innate immune system, the avian adaptive immune system is highly targeted and associated with the generation of immunological memory (34). The avian adaptive immune system is generally divided into two different responses: cell-mediated responses aimed at the clearance of intracellular pathogens and humoral immune responses targeting extracellular pathogens (35).

Cell-mediated responses are primarily actioned by T cells that develop within avian species in a manner strikingly like that seen in mammals. The thymus is initially colonized by mesodermal hemotopoietic cells during embryonic development, with these progenitor T cells undergoing differentiation and T-cell receptor (TCR) gene rearrangement; prior to migration to the peripheries (45). Antigen recognition is facilitated by TCRαβ, which is a heterodimeric surface receptor composed of two immunoglobulin superfamily domains. These domains combine to form TCRαβ heterodimers. In chickens, the two T-cell lineages can be distinguished based on the types of chains they possess, which include αVβ1 and αVβ2 chains. T cells expressing αVβ1 chains from infected or vaccinated chickens, but not naïve birds, recognize peptides from avian viruses in association with classical MHC molecules (46). Thus, T cells expressing αVβ1 chains are part of adaptive immunity. However, it remains unclear whether T cells expressing αVβ2 chains are also part of adaptive immunity, as no specific antigens recognized by this TCR have yet been identified (46). Moreover, the CD4 and CD8 co-receptors, which bind to major histocompatibility (MHC) I and MHC II, respectively, have also been identified in chickens and allow for further classification. However, unlike in mammals, a clear distinction of cell populations into cytotoxic and helper cells has not been demonstrated, but evidence suggests that both conventional CD4^+^ and CD8^+^ cells and innate-like T cells, such as γδ T cells, are involved in the control of pathogens via cytotoxicity or production of cytokines (46–52).

Like mammals, humoral responses in chickens are mediated by B cells. However, the origin of B cells is unique to birds, as they develop in the bursa of Fabricius. Akin to the development of T cells, the bursa is colonized by lymphoid precursors during embryonic development, before migration to the periphery. B cells generate antibodies specific to the encountered pathogenic threats, which requires the development of a diverse antibody repertoire. In mammals, this typically occurs through immunoglobulin (Ig) gene rearrangement; however, while this still occurs in birds, the primary way in which diversity is generated is through somatic gene conversion (53). While Ig gene rearrangement occurs continuously within the bone marrow of mammals, somatic gene conversion occurs entirely during the period of embryonic development in avian species (54). The activation of B cells within the periphery of chickens is identical to that reported in mammals, with the binding and processing of antigens by the B cell receptor followed by activation by helper T cells inducing CD40 activation. Chicken B cells can then differentiate into plasma cells, allowing the secretion of Ig that is capable of binding to and opsonizing extracellular pathogens (53). This allows the generation of long-term protective responses following vaccination, with increased Ig production following a secondary challenge (55).

### 2.3 Innate immune memory

The innate immune system evolved more than 600 million years ago in early eukaryotic cells to preserve cellular integrity and resist exogenous assault (56). The adaptive immune system is comparatively new, developing in the first jawed vertebrates approximately 450 million years ago (57). However, the adaptive immune system or ortholog has not evolved in invertebrates and plants, yet many of these organisms can survive for decades despite numerous encounters with rapidly evolving bacterial threats. Organisms lacking an adaptive immune system have also been observed to respond more effectively to secondary pathogenic exposure, with green bean plants and mealworm beetles demonstrated to resist reinfection following immunization with heat-attenuated bacterial pathogens (58–60). Furthermore, these immune memory responses in invertebrates and plants contrast with those typically seen within the vertebrate adaptive immune system, protecting against both homologous and heterologous secondary challenges, rather than being highly specific (61). These observations have led to a closer examination and eventual revaluation of the role of memory within the innate immune system of vertebrates.

**Figure 1:**
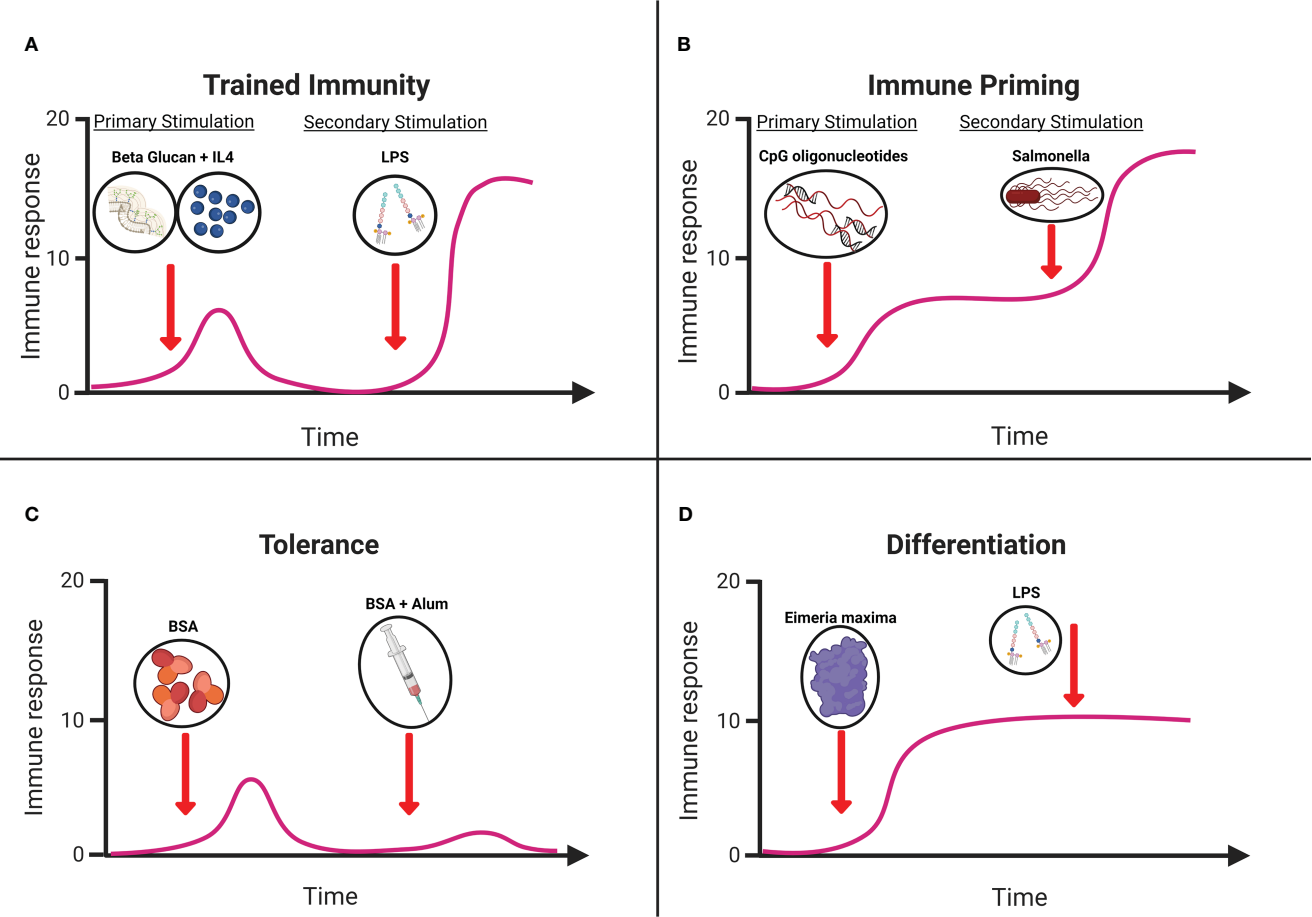
Graphical representation of **(A)** trained immunity. Treatment of primary chicken monocytes with Beta glucan microparticles and IL-4 significantly increased immune responsiveness to LPS (64). **(B)** Immune priming. Immunization with CpG oligonucleotides protecting chickens against Salmonella *in vivo* (65). **(C)** Tolerance. Dietary supplementation of bovine serum albumin (BSA) reduces immune responsiveness. **(D)** Differentiation. *In vitro* stimulation splenic dendritic cells with *Eimeria maxima* antigens resulting in morphological and functional changes. Schematic adapted from (66) created with Biorender.com.

Early evidence suggests the existence of non-specific innate immune memory in vertebrates, centered on historic trends of significantly reduced heterologous infections following the introduction of smallpox and Bacillus Calmette-Guerin (BCG) vaccines in human populations (62). As non-specific protection is atypical for the immunological memory of the adaptive immune system, innate immune memory has been suggested as the probable cause. This culminated in the identification of four distinct memory profiles within innate immune cells, particularly myeloid cells, including monocytes, granulocytes, dendritic cells, and macrophages (63). These four phenotypes were trained immunity, priming, tolerance, and differentiation. Trained immunity (**Figure 1A**) is defined as a functional adaptive program within innate immune cells brought on by a change in the activation state following exposure to a primary stimulus resulting in epigenetic modifications that persist despite a return to basal activation levels after removal of the stimulus. Epigenetic modifications result in enhanced responses following reactivation via homologous or heterologous stimuli with increased cell functionality and/or gene expression (66). Meanwhile, priming (**Figure 1B**) showed a similar change in the functional state within immune cells following stimulation, but this did not result in a return to baseline prior to secondary activation. Ultimately, this results in an augmented response to the subsequent challenge, which can be cumulative or synergistic (66). Tolerance (**Figure 1C**), on the other hand, is characterized by a reduction in the responsiveness of immune cells to a secondary challenge following a change in functional state by primary stimulation. This is typified by reduced metabolic activity and silencing of genes associated with inflammation (67). The final memory profile associated with innate cells is differentiation (**Figure 1D**), a long-term change in a cell’s functional state when a naïve immune cell becomes mature (66). Targeting this underutilized functionality of the innate immune system may lead to therapies with the potential to protect the host from a range of pathogens (62). This potential broad-spectrum protection is of great interest to the poultry industry as a management strategy for infectious disease in the post-antibiotic era.

## 3 Potential innate immune modulators

The advances we have made in our understanding of how the innate immune system works and its ability to improve through ‘training’ opens a clear opportunity to use it as a means of improving performance in animal husbandry. The ability to generate effective protection against a wide range of pathogens through the application of non-antibiotic agents holds great promise. Here, we present compounds that are potentially suitable for use in the poultry industry **Table 1**.

**Table 1:** Summary of immune modulators and their effects.

Immune modulator	Effect on innate immune system	References
Prebiotics		
Mannanoligoasacchardes (MOS)	Increases goblet cell number size and density. Increase TLR2b, TLR4, IL-12p35, and IFN-γ expression within the GI tract.	(68–71)
β-1,4-Mannobiose (MNB)	Increased expression of antigen presentation, host defense, and interferon associated genes.	(72)
Inulin	Increased heterophil/leukocyte ratio. Increased heterophil phagocytic activity.	(73)
Galactooligosaccharides (GOS)	Increased leukocyte oxidative potential.	(73)
Probiotics		
*Lactobacillus reuteri*	Increased intestinal cell proliferation and goblet cell differentiation. Increased lysozyme expression. Increased villus height: crypt depth ratio	(74, 75)
*Enterococcus faecium*	Increase intestinal cell proliferation. Reduction in apoptotic intestinal cells.	(76)
*Saccharomyces boulardii*	Increased HD11 phagocytosis and bactericidal activity. Down regulates proinflammatory cytokine responses	(77)
*Bacillus subtilis*	Increased proinflammatory cytokine production Reduced *Eimeria* spp. and *Clostridium perfringens* antibody production.	(78)
Adult chicken derived microbiota	Increased of IL-2Rα^+^ and activated NK cell numbers.	(79)
Beta glucan		
	Increased goblet cell numbers. Reduction in *Salmonella*, *E. coli*, and *Campylobacter* colonization. Induction of trained immunity response. Increased expression of proinflammatory cytokines. Increased heterophil phagocytosis, antibacterial activity, and oxidative burst capacity. Increased macrophage phagocytic activity and proportion of CD4 and CD8 positive lymphocytes. Increased peripheral blood mononuclear cell proliferation.	(80–86)
Pathogen associated molecular patterns (PAMPS)		
Cytosine-phosphorothioate-guanine oligodeoxynucleotides (CpG ODN)	Increased survival following *E. coli* and *S. typhimurium* challenge. Reduced *S. enteritis* visceral organ colonization. Significantly enhanced heterophil degranulation and oxidative burst. Induces shift in innate immune cell metabolic profiles.	(65, 87–93)
Lipopolysaccharides (LPS)	Increased peripheral blood monocyte phagocytosis, and nitric oxide and IL-1β production. Inhibition of dexamethasone immune suppression.	(94, 95)
Microbial metabolites		
Deoxycholic acid (DCA)	Reduced clinical histopathology and weight loss following necrotic enteritis. Reduction of proinflammatory cytokine responses. Improved protection against *C. jejuni* infection.	(96–99)
Butyrate	Increased expression of host defense peptides. Increased monocyte antibacterial activity against *E. enteritis.* Protection against *S. enteritis* challenge *in vivo*. Reduced necrotic enteritidis lesions. Attenuated *S. enteritis* adhesion to chicken enterocytes.	(100–102)
Propionate	Increased antimicrobial peptide expression. Reduction in *S. enteritidis* colonization	(103)
Vitamins		
Vitamin D	Reduction in proinflammatory responses to LPS. Reduced proinflammatory cytokine production. Increased macrophage nitric oxide production. Enhanced phagocytosis, chemotaxis, and microbial killing in monocytes *ex vivo*	(104–108)
Vitamin A	Deficiency associated with impaired immune function. Increased heterophil phagocytosis. Modulation of inflammatory cytokine expression.	(109–115)
Vitamin E	Increased phagocytosis of opsonized sheep red blood cells. Reduction in mortality and airsacculitis in Turkeys following *E. coli* challenge. Increased heterophil: lymphocyte ratio. Improved egg laying performance and decreased mortality following *S. enteritidis* challenge	(116–118)
Vitamin C	Improved physiological barrier integrity. Enhanced heterophil bacterial killing. Reduced *S. enteritidis* liver colonization following challenge. Reduced *S. enteritidis* colonization of the crop and caecal tonsils.	(119–122)
Plant derived compounds		
Carvacrol, cinnamaldehyde, and capsicum oleoresin	Increased lipid metabolism.	(123)
Thymol	Increased trans-epithelial electrical resistance. Increased blood phagocyte engulfment of microspheric hydrophilic particles.	(124)
Cranberry extract	Increased heterophil antibacterial activity and phagocytosis of *S. aureus*.	(125)
Ethanolic sea buckthorn extract	Increased layer peripheral blood monocyte phagocytosis.	(126)
Milk thistle, turmeric, shiitake mushroom extract, reishi mushroom extract, and cinnamaldehyde	Increase chicken spleen lymphocyte proliferation. Increased nitric oxide and proinflammatory cytokine production in HD11 cells.	(127, 128)
Hesperidin and genistein	Increased lymphocyte population within intestinal epithelium.	(129)

### 3.1 Prebiotics and probiotics

Prebiotics are indigestible carbohydrates that selectively promote the growth or activity of one or more bacterial species within the GI tract of the host to improve host health (130). Fructooligosaccharides (FOS), galactooligosaccharides (GOS), and mannanoligoasaccharides (MOS) are the most frequently utilized carbohydrates because of their inability to be broken down by vertebrate GI but can be metabolized by members of the microbiota (131). Prebiotics represent a viable alternative to growth-promoting antibiotics to improve growth, likely because of enhanced development of the avian innate intestinal barrier, resulting in improved nutrient uptake as well as innate responses. The innate intestinal barrier is key in host defense against infectious diseases, containing a range of cell types that directly contribute to host defense, such as enterocytes and Paneth cells which secrete antimicrobial peptides and prevent colonization of the intestinal surface by pathogenic bacteria. Moreover, the lamina propria within the epithelium contains antigen-presenting dendritic cells that facilitate crosstalk between innate and adaptive immune systems (132). MOS administration has been observed to modulate the innate immune system within the avian gut, increasing the goblet cell number, size, and density (68–70). Furthermore, prebiotic administration has also been shown to modulate innate effector cell responses as inclusion of MOS in broiler diets increased TLR2b, TLR4, IL-12p35, and IFN-γ expression within the GI tract (71). β-1,4-Mannobiose (MNB) supplementation of broiler diets also results in an increase in antigen presentation, host defense, and interferon-related genes (72). The *in ovo* delivery of inulin also increased the heterophil/leucocyte ratio and phagocytic ability at days 21 and 35 post-hatch. Additionally, the same study observed that *in ovo* administration of a GOS-based commercial prebiotic also increased leukocyte oxidative potential (73). Collectively, this suggests that prebiotics can effectively enhance the innate immune response within the avian GI tract, likely improving host responses to bacterial challenge, which, combined with the ability of prebiotics, inhibits pathogenic bacterial colonization of the GI tract through direct binding to pathogens or competitive exclusion (133).

Probiotics are live microorganisms that can confer health benefits to the host if administered in adequate amounts (134). The administration of probiotics can prevent infection through several mechanisms, including maintaining intestinal microflora, altering metabolism by enzymatic modulation, improving feed conversion, and stimulating the immune system (135). Several studies have identified an association between the microbiota and burden of pathogenic bacteria, with an increased *Campylobacter* load in the ceca of broiler chickens correlating with increased *Enterobacteriaceae* and reduced *Lactobacillus* abundance, respectively (136). This highlights the importance of microbiota composition and its effects on the burden of bacterial diseases in poultry. Therefore, the application of pre- and probiotics, aims to modulate microbial communities to improve performance and prevent disease.

One way in which probiotics may contribute to the modulation of the innate immune system is through the enhancement of epithelial barrier function, with oral gavage of *L. reuteri* in young chicks, resulting in enhanced intestinal cell proliferation and promotion of differentiation into mucin-producing goblet cells, likely due to the activation of Wnt/β-catenin and Mucin 2 expression, respectively. These effects, along with enhanced lysozyme expression following *L. reuteri* administration, suggest an improved innate barrier function in the intestinal tract following probiotic treatment (74). Administration of *E. faecium* to broiler chicks has also been shown to induce intestinal cell proliferation and reduce the number of apoptotic cells (76). However, this effect may vary depending on the phenotypic profile of the microbe, and a later study observed that oral administration of *L. reuteri* enhanced the villus height: crypt depth ratio but did not increase goblet cell numbers or mucin 2 expression (75). Pre-stimulation of the HD11 chicken macrophage-like cell line with the yeast probiotic *Saccharomyces boulardii* has also been observed to significantly increase phagocytosis and bactericidal activity against *C. perfringens*. Notably, pre-stimulation downregulates *C. perfringens-*induced pro-inflammatory responses, reducing the expression of IL-6, IL-10, TNF-α, and inducible nitric oxide synthase (iNOS) (77). Together, these results suggest an enhanced innate antimicrobial response, and a simultaneous reduction in inflammatory responses, which may be detrimental to bird performance and health. Dietary supplementation with *B. subtilis* has also resulted in immunomodulation in broiler chickens, significantly increasing pro-inflammatory cytokine production but greatly reducing antibody levels specific to *Eimeria* spp. and *C. perfringens* following challenge (78). These changes may suggest a shift in the immune response away from adaptation to the innate immune response following *B. subtilis* supplementation.

The role of gut microbiota in health and disease has been further elucidated in recent years, with dysbiosis and disruption of the gut microbiota linked to increased disease risk. The importance of microbial crosstalk within the gut–lung axis in mammals has also been observed, with disruption resulting in increased susceptibility to respiratory infections (137). Furthermore, the application of probiotics to modulate the gut microbiota has been shown to provide protection against respiratory diseases in humans, blocking the COVID-19 virus from entering and proliferating within host cells, as well as suppressing inflammatory responses (138). Further research is necessary to confirm the presence of the same axis within poultry, but evidence suggests that manipulation of the bird microbiota also has the capacity to directly modulate the innate immune system. This was clearly demonstrated with the inoculation of newly hatched broiler chickens with a microbial cocktail derived from adult birds, resulting in higher numbers of IL-2Rα^+^ and activated NK cells within the intestine at days 3 and 35, respectively. Increased NK cell activation was also observed in the blood and spleen of birds inoculated with adult-derived microbiota (79). This suggests that the composition of the microbiota has a significant impact on immunity in young birds and could be a valuable target for probiotic applications in the future.

Probiotics have historically been associated with improvements in gut health, along with recent developments in elucidating the role of the gut microbiota in the prevention of diseases within other organ systems, such as the respiratory system (137). Moreover, the microbiota has also been implicated in the development of immunity, particularly its role in the host response to vaccination in humans. It has been observed that microbiota composition in early life is associated with responsiveness to immunization (139, 140). This may be the case in poultry, with antibiotic depletion of the microbiota resulting in impaired IgM and IgY antibodies, and IFN-γ responses to H9N2 vaccination, with proper immune response restored following fecal microbial transplantation (141). However, further research is required to determine the capacity of the poultry microbiota to influence immunity to bacterial vaccination.

### 3.2 Beta-glucans

Beta-glucans are heterologous glucose-based polysaccharides produced by many fungi, bacteria, and plants. The pharmacological potential of the β- (1–3)-linked β-D-glucopyranosyl backbone and varying β- (1–6)-linked side chains have been extensively studied (142). Beta-glucans from a variety of sources have been tested for their efficacy in improving avian health and response to infection. Several studies have highlighted the benefits of dietary β-glucan supplementation in the enhancement of growth and nutrient digestibility in farmed animals, including pigs (143), cattle (144), and fish (145). In poultry, dietary supplementation with *S. cerevisiae* beta-glucan polysaccharide has been demonstrated to result in significant improvement in chick weight gain and improved FCR (80, 146–150). Beta-glucan supplementation also increases goblet cell numbers within the jejunum following *Salmonella* Typhimurium infection (82). This suggests that β-glucan may contribute to the maintenance of an effective gut barrier, which is critical for health and disease (151). Beta-glucan use has also been demonstrated to help prevent bacterial infection, with the inclusion of purified beta-glucan in the feed of white-leghorn chickens significantly reducing invasion and colonization of visceral organs by *Salmonella* Enteritidis in young chickens compared to a diet without the beta-glucan ration (83). Supplementation of the turkey diets with commercial β-glucan feed additives also resulted in reduced *E. coli* colonization in the air sacs and livers after challenge at one week of age with and without the birds undergoing transport stress (84). Continuous Beta-glucan supplementation of the turkey diets at 1 during the 16-week rearing period also significantly reduced the isolation of *Salmonella* and *Campylobacter* from the caeca of birds exposed to transport stress (81). This improved responsiveness to bacterial challenge is suggested to be a result of the ability of beta-glucan to bind to and interact with white blood cells such as macrophages via the complement receptor 3 and dectin-1 receptors, modulating immune responses (152). Activation of dectin-1 receptors has been demonstrated to amplify toll-like receptor (TLR) responses, including the production of cytokines and reactive oxygen species (ROS) and promoting phagocytosis (153, 154). Beta-glucan microparticles have been shown to induce the development of a trained immunity phenotype in primary chicken monocytes when co-administered with interleukin-4 (IL-4). After secondary stimulation with LPS, trained broiler and layer BMDMs showed significantly increased NO production and surface expression of colony-stimulating factor 1 receptor (CSF1R), CD40, and major histocompatibility complex class II (MHC-II) (64, 155). This increased NO response after secondary stimulation aligns with the trained immune phenotype observed in mammalian models (156). This highlights the potential of β-glucan to act as a modulator of innate immunity in poultry. *In vivo*, dietary supplementation with β-glucan in one-day-old chicks has also resulted in altered cytokine profiles in peripheral blood serum and increased expression of the pro-inflammatory factors interleukin (IL)-1, IL-2, interferon (IFN)-γ, and tumor necrosis factor-alpha (TNF-α) (80), factors associated with inflammatory responses to infections. The direct effect of dietary supplementation on the functionality of innate immune effector cells was investigated by isolating heterophils. This showed that heterophiles isolated from β-glucan-fed chickens had significantly increased phagocytosis, antibacterial activity, and oxidative burst capacity (83). In addition to heterophils, beta-glucan supplementation of one-day-old broiler chicks resulted in increased macrophage phagocytic activity and proportion of CD4 and CD8 positive lymphocytes (85). Modulation of innate effector cell functionality was also observed in turkey poults following dietary supplementation with commercial beta-glucan supplement. Isolation and examination of peripheral blood leukocytes at three weeks of age revealed an increased level of heterophils in the peripheral blood, with increased oxidative burst potential (84). Furthermore, dietary supplementation with 1% sophy β-glucan in Peking ducks significantly increased the proliferation of peripheral blood mononuclear cells in a dose-dependent manner (86), suggesting increased cellular immunity.

### 3.3 Pathogen associated molecular patterns

Bacterial, viral, fungal, and parasitic pathogens contain various components that are recognized by and stimulate immune cells. Vaccination with specific antigens has proven to be effective as a means of generating protection. However, in recent years, there has been increasing research into how stimulation of the innate immune system by PAMPs elicits a protective immune response. Single-stranded DNA PAMP Cytosine-phosphorothioate-guanine oligodeoxynucleotides (CpG ODN) have been identified as potent immunostimulatory molecules, with potential utility in cancer immunotherapy (157, 158), vaccine adjuvants (159), and mucosal vaccination (160). In poultry, immunostimulation with synthetic CpG ODN has been demonstrated to confer protection against bacterial infections. Subcutaneous or intramuscular administration of 10 ug or 50 µg to 22-day old broiler chicks significantly improved the survival of birds after *E. coli* challenge at 25 days post-hatching (87). Furthermore, intramuscular administration of 50 µg CpG-OGN to neonatal broiler chicks also significantly improved survival following virulent *E. coli* and *Salmonella* Typhimurium challenge (65, 88, 89). Treatment also resulted in a dramatic reduction in the frequency with which bacteria were isolated in CpG OGN-treated birds compared to the untreated group (65, 87–89), as well as a significant reduction in clinicopathological presentations following infection (65, 87, 89). Similar findings have also been reported in newly hatched birds treated with intraperitoneal administration of CpG ODN. Here significantly reduced colonization of visceral organs was observed in treated chicks following *Salmonella enteritidis* challenge (90). Needle-free intrapulmonary administration of 4 mg CpG-ODN per chamber between 6 h and five days before *E. coli* challenge was also demonstrated to reduce clinical presentations, frequency of bacterial isolation, and mortality without affecting the growth of birds (91). The efficacy of protection induced by CpG ODN was enhanced by formulation with carbon nanotubes and lipid surfactant delivery systems, which further reduced mortality and clinical presentations of colibacillosis disease (92). However, owing to the scale of commercial poultry farming, individually administered immunization is of little use. This has led to investigations into alternative routes, such as *in ovo* delivery, which can be achieved on a larger scale. Indeed, *in ovo* immunization with 50 µg of CpG ODN on day 18 of incubation significantly improved protection against *E. coli* or *Salmonella typhimurium* infection two days post hatch. This also reduces frequency of bacterial isolation from the air sacs of birds (88, 89). The protection rate against post-hatch *E. coli* challenge was further improved by *in ovo* immunization with the formulation of CpG OGNs with carbon nanotubes or lipid surfactants, without causing any adverse effects (92). Furthermore, *in ovo* administration of CpG ODNs significantly improved protection against an experimental model of colibacillosis yolk sac infection, which is a significant cause of early mortality in chicks (93). The mechanism involved in the protective effect of CpG ODN administration may be its ability to modulate the innate immune system responsiveness, which is characterized by significantly enhanced degranulation and oxidative burst responses in heterophils (90). Moreover, CpG ODN-induced immune responses have also resulted in a shift in metabolic profiles in innate immune cells, greatly upregulating glycolysis and fatty acid synthesis while downregulating ketogenesis and fatty acid β-oxidation (65). These metabolic shifts are strikingly like those observed in metabolic remodeling after the induction of trained immunity. Under these conditions, trained immune cells display significantly enhanced glycolysis, increased fatty acid metabolism, and oxidative phosphorylation pathways, leading to the promotion of epigenetic changes involved in proinflammatory responses (161). In fact, protection against *E. coli* meningitis in a murine model by intraperitoneal administration of CpG ODN has been cited as evidence for trained immunity in mammals (63, 162), suggesting a likely role for the innate program within the protection observed within poultry.

The use of PAMPs as immunostimulants for the induction of protective immunity in poultry has also been investigated. Lipopolysaccharides (LPS) form an important part of the outer membrane of gram-negative bacteria and are recognized by TLR4, resulting in a potent inflammatory response and the induction of cytokine expression (163). LPS from the plant symbiotic bacterium, *Pantoea agglomerans*, commonly found in edible plants (164), has been tested to prime the innate immune system against infection. Previously, its use in invertebrates was shown to improved phagocytic responses in shrimp (165) and carp (166). Oral administration of 10 ug/kg body weight/day LPS to broiler chicks significantly reduced their mortality within the first 10 weeks of age. Furthermore, analysis of the functional abilities of peripheral blood monocytes from birds administered LPS showed an increase in their phagocytosis, as well as NO and IL-1β production after stimulation with heat-killed *E. coli* (94). *P. agglomerans* LPS also inhibited dexamethasone-induced immunosuppression, cell death in thymic and bursal lymphocytes, and significantly increased the relative weight of both organs. This treatment also increased antibody production after *S. enteritidis* vaccination compared with that in control birds (95).

### 3.4 Microbial metabolites

The microbiota has been identified as inextricably linked to the development and function of the immune system, with dysbiosis associated with improper immunological responses in mammals (167) and chickens (168). Microbiota has been shown to communicate with the immune system, particularly at mucosal surfaces, through the production of a variety of molecules, often referred to as metabolites (169). These metabolites include short-chain fatty acids (SCFAs), secreted proteins or peptides, organic acids, biosurfactants, flavonoids, and vitamins (170).

Broadly defined as exogenously or endogenously generated intermediates or products of metabolism, metabolites can modulate the vertebrate immune system (169). Bile acids, known as secondary bile acids (SBAs), are host-derived molecules that undergo microbial transformation by members of the gut microbiota and gain novel functions (171). Two of these SBAs, deoxycholic acid (DCA) and lithocholic acid (LCA), are the most abundant metabolites within the human microbiome (172), as well as important immunomodulators (171). In poultry, multiple studies have demonstrated that dietary supplementation of one-day-old broiler chicks with DCA reduced clinical histopathology in the ileum and the resultant weight loss following *Eimeria maxima* and *C. perfringens-*induced necrotic enteritis (NE). These phenotypic changes following DCA supplementation in chickens, were likely due to reduced *C. perfringens* and *E. maxima* colonization in the ilium (96, 97). DCA alone inhibited *C. perfringens* growth *in vitro* (97); however, DCA supplementation resulted in a reduced pro-inflammatory innate response to NE, including lower expression of IFN-γ and MMP9 cytokines (96), Lipopolysaccharide-Induced TNF Factor (LITAF), and Cyclooxygenases-2 (COX-2) (97). This attenuation of the inflammatory response may contribute to reduced clinical signs after NE infection. Supplementation of day-old broiler diets with DCA has also been effective at protecting against *C. jejuni* infection, preventing colonization, and improving bird performance following infection (98). Interestingly, DCA had no impact on *C. jejuni* viability *in vitro*, suggesting that modulation of the microbiota contributes to protection. Similar results have also been observed in mice, with oral DCA administration resulting in the prevention of *C. jejuni* colitis. However, we observed that DCA inhibited proinflammatory gene expression of IL1β, Cxcl2, and IL17a, but also led to downregulation of the mammalian target of rapamycin (mTOR) pathway within primary splenocytes following exposure (99). This suggests a potential modulatory effect of DCA on innate cells and correlates with a reduction in proinflammatory responses (173). Furthermore, mTOR downregulation has also been associated with enhanced antimicrobial activity in macrophages, with exposure of primary *murine* monocytes to the short-chain fatty acid butyrate during differentiation, resulting in significantly increased bacterial intracellular killing, alongside decreased mTOR (174).

Organic acids have been frequently use as food preservatives and exert antimicrobial effects on the growth of pathogenic bacteria (33). Many organic acids, particularly short-chain fatty acids (SCFAs), are produced by the fermentation of undigested carbohydrates by members of the gut microflora. Some of these organic acids have been included in animal feed and function as fungistats (33). However, there is growing evidence to suggest that SCFAs may have a potent immunomodulatory effect, with butyrate being associated with enhanced host defense peptide (HDP) expression in the HD11 chicken macrophage cell line, primary chicken monocytes, and intestinal explants. Furthermore, chicken monocyte antibacterial activity against *S. enteritis* was seen to increase with butyrate exposure in a dose-dependent manner, with dietary supplementation of chicken feed with sodium butyrate resulting in protection against challenge (100). Butyrate also significantly reduced the expression of pro-inflammatory cytokines IL-1β, IL-8, and MMP9 following *S. enteritis* challenge (101). Supplementation with butyrate attenuated NE-induced weight loss and reductions in FCR, as well as the severity of clinical lesions (102). Butyrate has also been shown to modulate the response of innate immune cells associated with physical barriers to infection, with exposure of primary chicken enterocytes to butyrate resulting in significantly reduced adhesion and invasion of *S. enteritis* (101).

Propionate is a three-carbon SCFAs produced by the *Bacteroidetes*, *Firmicutes*, and *Lachnospiraceae* families within the GI tract (175). Dietary supplementation with sodium propionate has been demonstrated to dramatically alter the composition of the cecal microbiota and inhibit fat deposition within broilers by downregulating fat synthesis genes and affecting feed consumption (176). Moreover, dietary supplementation with propionate associated with chromium has been observed to significantly improve final body weight, weight gain, feed efficiency, and carcass characteristics in broilers compared to control diets (177). In mammals, propionate has been shown to inhibit *S. typhimurium* colonization, likely through the inhibition of growth and modulation of intracellular pH (178). There is also extensive evidence in mammals suggesting that propionate has immunomodulatory properties. This includes the inhibition of inflammatory responses following stimulation with *S. aureus in vitro* and *in vivo*, with murine macrophages pre-incubated with propionate demonstrating reduced nitric oxide production and decreased pro-inflammatory cytokine production in mice treated with propionate following *S. aureus* challenge (179, 180). Propionate also modulates AMP expression in pig intestinal epithelial cells and macrophages (103). Indeed, this has also been observed in poultry exposed to chicken HD11 cells and primary monocytes exposed to propionate, resulting in significantly increased *AvBD9* and *cathelicidin B1* expression. Moreover, supplementation with propionate resulted in significant reductions in cecal *S.* Enteritidi*s* following the challenge of four-day-old broiler chicks (103).

### 3.5 Vitamins

Vitamins, which are essential organic compounds, can also be produced by the microbiota or attained through the diet. Several vitamins have been found to be crucial for immunological functions of immune cells including macrophages and lymphocytes (181). The effects of dietary vitamins on the immune system of poultry have been reviewed by Shojadoost et al., who focused on the effects of vitamins on the innate immune system.

Vitamin D has been identified as a key contributor to innate immunity, promoting antibacterial responses, and pattern recognition receptors (PRR) and cytokine gene expression in humans (182). In poultry, supplementation with active vitamin D_3_, 25-hydroxycholecalciferol (25-OH-D3) improved survival and ameliorated inflammatory stress (104, 183, 184). Furthermore, a dietary deficiency of active vitamin D_3_, 25-hydroxycholecalciferol (25-OH-D3), reduced the performance and egg quality in laying hens, and this condition was resolved with 25-OH-D3 supplementation (104). Vitamin D_3_ supplementation significantly improved FCR in broiler chicks (185), increased villus length/crypt depth ratios within the first 21 days of growth in broiler duodenum and jejunum (186), and significantly improved body weight following LPS challenge (105). Collectively, these studies suggested that 25-OH-D3 is important for poultry productivity. There is also great interest in the ability of 25-OH-D3 to act as an anti-inflammatory modulator. 25-OH-D3 supplementation reduces pro-inflammatory responses following LPS challenge. This included a reduction in pro-inflammatory IL-1β and IL-6 expression, as well as the IFN-γ:IL-4 ratio after LPS challenge (104, 105). It is hypothesized that modulation of the NF-κB pathway contributes to the effects of 25-OH-D3 (104). Similarly, inhibition of proinflammatory cytokines, such as IL-1β and CXCL8, was observed in the MQ-NCSU chicken macrophage cell line pre-treated with 25-OH-D3 (106). Furthermore, pretreatment with 25-OH-D3 also increased NO production from macrophages after LPS or Pam3-CSK stimulation. Moreover, 25-OH-D3 reduced the inflammatory response of chicken T lymphocytes but did not affect their degranulation response (107). Modulation of innate effector cells after dietary 25-OH-D3 supplementation in broilers was observed, as demonstrated by improved *ex vivo* monocyte phagocytosis, chemotaxis, and bacterial killing (108). Together, the improvement in innate immune cell function may confer protection against a range of bacterial infections and reduce immunopathology, which can reduce productivity by reducing tissue damage (187) in poultry. However, further investigations are needed to determine the effectiveness of 25-OH-D3 supplementation in preventing or reducing bacterial infections.

After hatching, the inclusion of Vitamin A group of fat-soluble micronutrients, inhibits production losses associated with vitamin A deficiency, which is known to cause poor growth, reduced egg production, and impaired immune development and function (188, 189). The inclusion of vitamin A also improved performance, as measured by significantly higher body weights observed in broiler chickens supplemented with the standard (1,500 IU/kg) and high 1(5,000 IU/kg) vitamin A-supplemented diets compared to unsupplemented and pair-fed controls (190). Vitamin A supplementation also improved the wing weight of broilers in a dose-dependent manner. This regime also reduced the occurrence and severity of wooden breasts and white striping within meat (191). In mammals, vitamin A has been linked to the development of physiological barriers, and its deficiency results in impaired respiratory, gastrointestinal, and urogenital mucosal barrier function, which is partially attributed to the loss of mucus-secreting goblet cells (192). In poultry, vitamin A deficiency decreases respiratory mucin and IgA production (109) and gastrointestinal goblet cell proliferation (110), and this condition is associated with the development of squamous metaplasia and epithelial damage in turkeys (111). Taken together, these results suggest that the loss of the protective mucosal barrier on epithelial surfaces increases the risk of infection (189, 192). Vitamin A is also essential for the development of innate immune effector cells, and its deficiency diminishes the ability of mammalian phagocytes to engulf and lyse bacteria after infection (192). In poultry, vitamin A deficiency significantly reduced phagocytosis of heterophils in broiler chickens at 49 days post-hatching. Furthermore, addition of vitamin A exceeding the recommended dosage increased the phagocytosis index of heterophils at 28- and 49-day post-hatch compared to both vitamin A-deficient birds and birds at the recommended dose (112). Vitamin A supplementation increased the phagocytosis index of chicken intra-abdominal macrophage isolates from white-leghorn chickens compared to those from vitamin A-deficient birds (193), suggesting a vital role for vitamin A in the functional abilities of immune cells. The presence of vitamin A has been linked to the regulation of inflammatory processes, and its deficiency upregulates the expression of pro-inflammatory cytokines and immunopathology in a rat model (192). Furthermore, pre-incubation of murine macrophages with vitamin A in the form of retinoids inhibits the expression of inflammation-associated IL-12 and IFN-γ following activation both *in vitro* and *in vivo* (115). Therefore, it was unexpected that the *in ovo* administration of 90 μmol/egg vitamin A in broiler chicks enhanced IL-12 and IFN-γ expression, suggesting a differential response in birds (113). However, an earlier study by the same group observed that the immunomodulatory effects of vitamin A were dose-dependent; *in ovo* administration of 90 μmol/egg retinoids upregulated IFN-α, IFN-γ, IL-1β, IL-2, IL-8, IL-12, and IL-13 in chicken embryos, but 270 μmol/egg downregulated these cytokines (114).

Vitamin E is a group of lipid-soluble molecules, well known for their antioxidant properties and ability to modulate immune cells, as they are found in higher concentrations in these cells than in other cells in the blood (194). Vitamin E exists in four functional forms α, β, γ, and δ, with α being the most abundant and functionally active form (189). Poultry feed supplementation is believed to improve bird performance, with higher broiler body weight and improved FCR observed each week for five weeks post-hatching (195). However, these findings were not confirmed in any of these studies (196, 197). Vitamin E deficiency greatly impairs the ability of the adaptive immune system to respond to infection and is associated with reduced antibody production and lymphocyte proliferation. Vitamin E deficiency results in increased inflammatory cytokine production and impaired antibacterial and phagocytic activities of neutrophils in infants (194). Furthermore, *in vivo* supplementation of excess vitamin E increased the phagocytic activity of rat alveolar macrophages (198), suggesting that vitamin E ability can enhance innate immune responses. In poultry, excess dietary vitamin E increased phagocytosis of opsonized sheep red blood cells by macrophages isolated from the abdominal exudate of three-week-old broilers. However, no such increase was observed in the phagocytosis of non-opsonized cells or in broilers at five- and seven-week post-hatch (116). Vitamin E has also been studied for its potential protective effect against bacterial infection. The administration of vitamin E to the drinking water of turkeys resulted in a reduction in mortality and airsacculitis following *E. coli* challenge as well as a reduction in bacterial colonization of the liver. The addition of vitamin E has also been associated with a shift in the heterophil-to-lymphocyte ratio, indicating an enhancement in innate responses (117). Moreover, vitamin E supplementation has been observed to improve laying performance and reduce mortality following *S. enteritidis* infections (118).

Unlike the other vitamins discussed above, vitamin C is water-soluble and synthesized from glucose. Moreover, vitamin C is not stored in the body; however excess dietary intake is excreted by the kidneys. Unlike humans, poultry can produce endogenously but require supplementation during times of stress (189). Indeed, vitamin C supplementation has been observed to improve poultry performance, egg weight, and quantity as well as enhance FCR and carcass weight in layers and broilers, respectively (199). Vitamin C is a potent antioxidant that functions as a cofactor for the enzymes involved in biosynthesis and regulation. Vitamin has also been observed to play a vital role in the function of the innate immune system, helping maintain barrier integrity, enhancing activity, acting as an electron donor for phagocytes, and modulating cytokine expression (119). Indeed, heterophils isolated from broilers had improved bacterial killing ability *in vitro* following vitamin C exposure, although no increase in phagocytosis was detected (120). This increased antimicrobial activity may be because of vitamin C on the production of reactive oxygen species (ROS) from heterophiles. This notion is confirmed by results showing that vitamin C deficiency reduces human neutrophils to produce ROS, which can be rectified by vitamin C supplementation (200). This suggests that vitamin C facilitates ROS production in the innate effector cells. Supplementation with vitamin C has also improved protection against bacterial infection, which is associated with a reduction in the translocation of *S. enteritidis* to the liver, maintaining intestinal morphology, and the composition of the cecal microbiota after infection. However, no significant reduction in mortality was observed in these animals (121). This is in accordance with the observation that prophylactic supplementation of vitamin C, for six days after hatching led to a significant reduction in *S. enteritidis* colonization in broiler crops after infection. Moreover, therapeutic administration of vitamin C, in one-day old broiler chicks significantly reduced bacterial colonization of the crop and cecal tonsils at three- and ten days post-infection (122).

### 3.6 Plant derived compounds

In human medicine, growing evidence suggests that plant extracts may have potent immunomodulatory potential, affecting neutrophils (201, 202), leukocyte function (203), and intestinal barrier function (204). This has also been observed in poultry, with intestinal intraepithelial lymphocytes isolated from broilers fed carvacrol, cinnamaldehyde, or capsicum oleoresin, demonstrating altered metabolic gene expression and a shift towards lipid metabolism (123). Additionally, dietary supplementation of day-old broilers with thymol significantly increased trans-epithelial electric resistance (124), suggesting reduced susceptibility to infection, as loss of membrane integrity is associated with bacterial pathogenesis (205). In the same study, thymol supplementation was also observed to increase isolated blood phagocyte engulfment of microspherical hydrophilic particles, indicating additional immunomodulatory effects on innate effector cells (124). Dietary supplementation with cranberry extract has also been demonstrated to significantly enhance chicken heterophil antibacterial activity, increasing phagocytosis, and intracellular killing of *S. aureus in vitro* (125). Meanwhile, enhancement of phagocytosis of both Indian ink and sheep erythrocytes was also reported in peripheral blood phagocytes isolated from laying hens following stimulation with ethanolic sea buckthorn extract. However, garlic extract diminished the phagocytic ability of these cells, suggesting an extract-dependent effect (126). Milk thistle, turmeric, shiitake, and reishi mushroom extracts, as well as cinnamaldehyde have all been demonstrated to significantly enhance the proliferation of chicken spleen lymphocytes as well as modulate cellular responses, increasing NO production and expression of pro-inflammatory cytokines in the HD11 cell line following stimulation (127, 128). Hesperidin and genistein, flavonoids derived from citrus and soy, respectively, also increased lymphocyte number within the intestinal epithelium following dietary supplementation of 21-day-old-broiler chicks (129).

## 4 Discussion

Bacterial diseases can have a devastating effect on poultry welfare and productivity in poultry rearing systems (206). Antibiotics have been widely used to treat or prevent bacterial infections and to promote growth in poultry farms. However, this use has led to the emergence of antibiotic-resistant bacteria, which can cause serious health issues in both humans and animals. It is crucial for the poultry industry to take proactive steps to reduce the use of antibiotics and use alternative strategies to control and treat bacterial infections. These factors, combined with the need to expand food production to accommodate the growing global population collectively highlight the potential value of effective manipulation of the avian innate immune system by immunostimulatory compounds. Here, we have highlighted several ‘classes’ of immunomodulatory compounds that can enhance poultry performance and immune responses to bacterial pathogenic challenges. The use of these compounds as either feed additives or through direct application *in vivo* or *in ovo* presents an attractive management strategy owing to the non-specific responsiveness and ubiquity of the innate effector cells and physiological barriers within birds (34). Thus, effective innate immunomodulation and/or induction of trained immunity within birds may provide effective protection against multiple distinct pathogens following the application of a single stimulant (207). The ability to protect against a range of pathogens is highly beneficial and difficult to achieve using traditional vaccination strategies (25). Moreover, the ability to induce protection against multiple bacterial pathogens may potentially facilitate a reduction in antibiotic use or a shift towards narrow-spectrum antibiotics, a key recommendation in poultry antibiotic stewardship programs (208).

The reported capacity of immunomodulators to improve bird performance, health, and welfare is also highly desired and reminiscent of the widespread use antibiotic growth promoters without the risk of AMR selection (209). Indeed, supplementation of prebiotics, such as *S. cerevisiae* derived MOS (210, 211) and beta glucan have been shown to be effective at increasing bird performance, leading to enhanced FCR and weight gain (80, 146–150). Several probiotics have also led to increased weight gain in birds following supplementation (212) in addition to plant bioactive compounds (213–215) and essential oils (216). Collectively, this suggests that the immunomodulators discussed may provide the combined benefit of inducing effective protection against bacterial infection, in addition to enhancing bird performance, resulting in greater yields. Additionally, the favorable modulation of the innate immune system may also reduce production costs, as currently within the UK, and broilers routinely receive over a dozen separate vaccine treatments within the first 24 days of life (217). These treatments, as well as additional vaccines that may also be included due to seasonal factors or pathogenic outbreaks, all present financial and labor costs to purchase and implement (217). Effective modulation of the innate immune system may provide protection against multiple vaccine targets, thereby reducing costs. The ability of immunomodulatory compounds to effectively impact bird health when included as dietary additives may also avoid potential issues associated with spray vaccination, such as sudden drops in body temperature during application at one-day post-hatch within hatcheries and poultry units (218). Moreover, there remains the potential for the repurposing of waste products from other industries as stimulants, such as the use of beta-glucans from spent yeast being extracted for technological applications (219). In addition to reducing the costs associated with the production of immunostimulatory compounds, this also increases sustainability and minimizes environmental impact. Owing to the non-antibiotic nature of the compounds examined, a limited environmental impact would also be likely due to the reduced presence of antibiotic compounds in animal-derived compounds and animal waste, reducing the likelihood of AMR selection within the local environment (220). Proper modulation of the innate immune system may also enhance vaccine effectiveness, increase responsiveness, and promote crosstalk between the innate and adaptive immune systems, which is an essential factor within effective vaccination (221).

However, despite the many potential benefits associated with manipulation of the innate immune system, a greater understanding of the potential side effects is required before widespread adoption. One central issue that may detrimentally impact immunomodulation in poultry is the disadvantageous induction of inflammation. Indeed, in mammals, the induction of trained immunity has been found to be strongly associated with atherosclerosis following training with both β-glucan and BCG (222, 223). Furthermore, inappropriate activation of trained immunity has also been suggested to be associated with the development of autoimmune and autoinflammatory conditions, which would be deleterious to bird welfare and performance if this occurs in poultry (224). It can also be postulated that the risk of adverse inflammatory responses may increase because of the intensive vaccination programs currently associated with poultry production. Thus, there is a need to investigate the effectiveness of immune stimulants in birds under a range of conditions, including different farming setups, birds that have undergone vaccination, and different bird breeds, to accurately evaluate their effectiveness *in situ*.

In summary, our review indicates that innate immune modulation could be a promising approach for controlling bacterial infections in poultry and for improving their performance. Based on previously published literature, we identified a range of compounds that have shown potential in enhancing the innate immune response to bacterial challenges. However, further work is needed to (i) determine whether this approach is effective against all bacterial infections in chickens, (iii) evaluate the efficacy and safety of using specific compounds to control bacterial infection, and (iii) explore the potential benefits and risks of combining these compounds with vaccines against bacterial infections to enhance vaccine effectiveness without inducing an inflammatory response leading to immunopathology in birds. The optimal compounds are those that control bacterial infections, improve growth performance and gut health barriers, and reduce the severity of inflammation.

## Author contributions

JA and SB contributed to the conception and structure of the review. JA performed the literature search and wrote the first draft of the manuscript. SB and JA wrote sections of the manuscript. All authors contributed to the article and approved the submitted version.
